# The impact of the COVID-19 pandemic on students’ feelings at high school, undergraduate, and postgraduate levels

**DOI:** 10.1016/j.heliyon.2021.e06465

**Published:** 2021-03-08

**Authors:** Claudia Camacho-Zuñiga, Luis Pego, Jose Escamilla, Samira Hosseini

**Affiliations:** a School of Engineering and Sciences, Tecnologico de Monterrey, Campus Toluca, CP 50110, Mexico; b Departamento de Inteligencia de Audiencias, Tecnologico de Monterrey, Campus Monterrey, NL 64830, Mexico; c Institute for the Future of Education, Tecnologico de Monterrey, Monterrey, CP 64849, NL, Mexico; d Writing Lab, Institute for the Future of Education, Tecnologico de Monterrey, Monterrey, CP 64849, NL, Mexico; e School of Engineering and Sciences, Tecnologico de Monterrey, Campus Monterrey, NL 64849, Mexico

**Keywords:** COVID-19 pandemic, Educational innovation, Educational policy, Public mental health, Students' feelings

## Abstract

The COVID-19 pandemic and the enforced restrictions have harshly affected educational sectors in 161 countries around the world. With more than 1.6 billion students away from normal school life, the crisis threatens the teaching and learning processes and the students’ emotional health. Herein, we present the result of a careful assessment of the feelings of over 13,000 students at high school, undergraduate, and postgraduate levels across 36 campuses over 8 subsequent weeks from the onset of the COVID-19 pandemic. The results indicate a general low energy level and dominance of negative feelings among the students regardless of their academic levels. We have recorded 5 responses (being *anxious*, *stressed*, *overwhelmed*, *tired*, and *depressed*) as the most frequently reported feelings in the time of lockdown. Overall, 14% of those who have reported to suffer from these feelings have also identified a need for professional help in managing their feelings throughout the quarantine period. The current study also presents several strategies to combat the undesirable consequences of COVID-19 pandemic.

## 1 Introduction

Feelings play a significant role in psychological well-being of students hence directly affecting all aspects of their academic lives (Health, 2016; Moeller et al., 2020; Phan et al., 2019). Specifically, positive feelings (e.g. enjoyment and interest) were found to be associated with students' attention, concentration, engagement, and persistence in learning activities which positively correlate with academic achievements (Eccles, 2005; Moeller et al., 2020; Schiefele, 1996). On the other hand, negative feelings (e.g. boredom, burnout, and anxiety) are known to diminish cognitive resources thus negatively impacting school performance and academic achievement (Madigan and Curran, 2020; Moeller et al., 2020; Samuel and Burger, 2019). The SARS-CoV-2 widespread challenged the normal human life around the globe in 2020 (Ng et al., 2020). With the goal of slowing the spread of the virus, governments of different countries implemented restrictions for reducing social contact over different dates and scales. Nevertheless, the COVID-19 pandemic has imposed a tremendous emotional burden on students at all academic levels and threatened their mental health (Gavin et al., 2020; Grubic et al., 2020; Pfefferbaum and North, 2020). Inevitably, universities and colleges across the world face unexpected challenges as students have manifested signs of anxiety and stress. While a certain degree of anxiety could be a rational response to uncertainties that revolve around a pandemic experience (XiangNg et al., 2020), different reports highlighted a sharp peak in prevalence of depressive and anxious symptomatology among students that ranges from moderate to severe (Grubic et al., 2020; Torales, O'Higgins, Castaldelli-Maia and Ventriglio, 2020; Wang et al., 2020; Zou et al., 2020). A study reported 73% low mood, 7% anxiety, and 17% anger for students under lockdown while a slim percentage of positive feelings (5%) was also recorded (Brooks et al., 2020). According to Cao et al., different levels of anxiety ranging from normal (~75%), to mild (~21%), and moderate (~3%) were recorded among students of Chinese college under quarantine due to COVID-19 widespread. A study among high school students revealed an overall report of stress (~29%), anxiety (~12%), and depression (~5%) (Moeller et al., 2020). In fact, American College Health Association and World Health Organization (WHO) have reported that, even in regular academic periods, heightened psychological distress and mental disorders associated with attrition are common among college students, particularly when they are temporarily away from their schools (Auerbach et al., 2016; Jarrett et al., 2012). It is, therefore, expected that the time of lockdown and uncertainty as a result of a global pandemic brings alarming consequences at individual and collective levels.

Cases of the COVID-19 were first identified in Mexico on February 28^th^, 2020. Twelve days later, following the official announcements of Mexican authorities and WHO, Tecnologico de Monterrey was the first higher education institution in Mexico to take preventive measurements to mitigate, delay, and minimize the escalation of the infection. Tecnologico de Monterrey cancelled in-person classes and suspended academic and student events across all different campuses, nationwide. Following this timely course of action, several initiatives were established through which the stakeholders (e.g. students, faculty members, and administrators) remained in active communication and performed their duties as expected. A task-force presented by Tecnologico de Monterrey was “Budy Academico” or *Academic Buddy* which allowed a smooth transitioning of the faculty and students to the online methods of teaching and learning throughout the first few weeks of the quarantine. Consequently, all activities migrated to a flexible digital plus model (MFD+), while, Tecnologico de Monterrey was actively involved in providing similar services to other universities across Latin America (LATAM). Integrating a number of cutting-edge educational technologies, including a learning management system (LMS), to generate hybrid and distance learning experiences, MFD + aimed at mimicking in-person classroom experience for the students and facilitating the teaching and learning process. While the peer-support provided by *Academic Buddy* program assisted faculty members in delivering their day-to-day tasks, the university unceasingly monitored the well-being of the students at different stages of the pandemic. To date, this continuous observation of physical, mental, and emotional health of the students is ongoing. Other initiatives such as “TQueremos” (*We Care for You*), "life@home", and “Cuida Tu Mente” (*Take Care of Your Mind*) were established to provide support to emotionally and mentally vulnerable members of Tecnologico de Monterrey.

This article presents the results of our weekly assessments of the students’ feelings over the course of 8 subsequent weeks from the onset of the quarantine in Mexico. The online questionnaire was distributed among high school, undergraduate, and postgraduate students across 36 campuses of Tecnologico de Monterrey accessible by 13,000 students. The open-ended survey allowed us to record a wide range of feelings and narrow the study down to the most alarming ones that may require professional support. The study concentrates on emotional valence and the energy levels of the students as two important measures to assess the emotional health of the students. Furthermore, to battle the detrimental impacts of COVID-19 pandemic on the emotional and mental health of the students, we propose a number of strategies and present the preliminary results of these implemented methods.

## 2 The impact of COVID-19 pandemic on students’ mental health

Apart from the physical health complications of COVID-19 pandemic, the pandemic has also imposed mental, emotional, and social challenges to our lives. Reports of the literature suggested that college students often experienced compounded negative emotions during the school closure (e.g. holidays or spring breaks) (Van Bortel et al., 2016; Zhai and Du, 2020) and even suffered from poor mental health due to the disruption of academic routine (Michael Agnew and Khan, 2019; Zhai and Du, 2020). Some students who found the campus homelike struggled with loneliness and isolation due to the disconnections from friends and partners (Zhai and Du, 2020). Previous studies have suggested that public health emergencies can have psychological effects on college students imposing anxiety, fear, and worry among others (Baloran, 2020; Cao et al., 2020; Mei et al., 2011). In addition, the students experienced uncertainty and sudden disruption of the semester and their activities, their research projects and internships, and delaying graduation. Since youth can be asymptomatic carriers, they have also worried about the chances of contracting the illness and transferring the infection to those around them or putting their older family members at increased risk of this deadly disease (Pan et al., 2010; Zhai and Du, 2020). Brooks et al. (2020) reported that fear of infection, duration of quarantine, frustration and boredom, inadequate supplies and information were great stressors during quarantine. The authors also suggested that the psychological impact of quarantine on the population was wide-ranging, substantial, and the consequences must be dealt with not only during the pandemic but also months or even years later (Brooks et al., 2020). For the students who were distant from their peers and were experiencing life in lockdown, the anxiety level has shown a gradual increase as this psychological disorder is more likely to occur and worsen in the absence of interpersonal communication (Cao et al., 2020; Kmietowicz, 2020; Xiao, 2020). Within the past few months, understanding of the emotional state of the general population and that of students has become the focus of research in order to find the best course of actions and public health decisions (Brooks et al., 2020; Cao et al., 2020; Zhai and Du, 2020). It is, therefore, imperative for universities and colleges to build awareness around students’ feelings and to empower them to effectively regulate their feelings and to seek help and support during this biological disaster.

## 3 Methodology

### 3.1 Data collection procedures

The data were collected on a weekly basis between March 13^th^ and May 8^th^, 2020, using an online survey. A total of 5,000 high school students (HSS), 5,000 undergraduate students (UGS), and 3,500 postgraduate students (PGS) were selected by randomly generated numbers matching the institutional student identification. The invited students were the representatives of all 36 campuses. Since the participation was voluntarily, the overall approximate participation was 5%–7%. With a 95% confidence level, these sample sizes give confidence intervals of 6–8% for HSS, 4–7% for UGS and 6–10% for PGS.

The personal information of the students was treated as strictly confidential. The research project was approved by the Institutional Research and Ethics Review Committee from the Office of the Vice President of Research and Technology Transfer from Tecnologico de Monterrey, and it complied with the principles of the Declaration of Helsinki for research on human subject. Table 1 shows the number of participants per week and their educational levels (see Table 2).

**Table 1:** Distribution of participants by educational level and week.

Educational level/Week	W1	W2	W3	W4	W5	W6	W7	W8	W1–W8
Postgraduate	182	272	195	169	125	103	105	111	1262
Undergraduate	453	227	296	233	176	212	205	319	2121
High school	263	194	215	141	122	142	153	243	1473
Total	898	693	706	543	423	457	463	673	4856

**Table 2:** The distributed questionnaire among students of different academic level throughout the 8 weeks of pandemic.

Number	Question	Choices
Q1	What was your level of energy this week?	Very low, low, neutral, high, very high
Q2	In one single word, how did you feel this week?	NA
Q3	How pleasant or unpleasant is this emotion that you felt?	very unpleasant, unpleasant, neutral, pleasant, very pleasant
Q4	In addition to the previous feeling, what other feelings did you have during this week? (Maximum four)	NA
Q5	You mentioned that your main feeling is unpleasant/very unpleasant, do you think that you need professional help to handle your emotions?	yes, no
Q6	Would you like to be contacted by a specialist from the “TQueremos” program to help you in handling your emotion?	yes, no
Q7	Have you heard about the program “Cuida tu Mente”?	yes, no
Q8	Have you heard about the program “life@home”?	yes, no
Q9∗	Have you participated in one of the “life@home” activities?	yes, no
Q10∗	In your opinion, how much has the "life@home" program contributed to your emotional health and mental well-being? (where 1 is equivalent to “it does not contribute at all to my mental well-being and emotional health” and 5 is equivalent to “it contributes a lot to my mental well-being and emotional health”)	1, 2, 3, 4, 5

∗ If the participant answered "yes" to Q8 they were guided to Q9 and Q10.

### 3.2 Survey

With respect to the mental health, RULER strategy (2016) (*recognizing* emotions, *understanding* the causes, *labeling* emotions with the right choice of words from a vast vocabulary corpus, *expressing* the emotions, and *regulating* them) represents an opportunity to assess the daily feelings of the students (Nathanson et al., 2016). Moreover, as Moeller et al. (2020) have demonstrated that the feelings can be expressed in the common daily language. We have based the development of our questionnaires on these two strategies. The questionnaire was piloted on 5,000 HSS, 5,000 UGS, and 3,500 PGS during the first week of the remote learning and was further improved based on the answers received during the pilot study. The survey was developed on Qualtrics and via the institutional access of Tecnologico de Monterrey. The survey link was disseminated through the institutional email and was sent to the selected candidates. Using the institutional Matrix number which is unique to each student avoided duplicated responses.

### 3.3 Statistical analysis

The energy level was assessed based on the answers provided to Q1. For the analysis of the feelings, the answers to Q2 and Q4 were categorized to discrete words mainly considering synonyms. For instance, the answers “muy cansado” (very tired), “agotado” (drained), and “exhausto” (exhausted) were all associated with “cansado” (tired). In order to understand the probability of the students requiring professional help to handle their feelings, the answers to Q5 (as binary variable, 0 = no and 1 = yes) were analyzed with a logistic regression. The logistic regression measured the relationship between the need for professional help (categorical dependent variable) and the explanatory or independent variables by estimating probabilities using a logistic function. Besides the main feelings, the explanatory variables of the analysis were the level of energy (Q1, numerical value: -2 = very low, -1 = low, 0 = neutral, +1 = high, +2 = very high), the educational level (HSS = 0, UGS = 1, PGS = 2), and the weeks under lockdown (1, 2, 3, …, 8) (Table 2). The obtained model was evaluated through the area under the curve (AUC) and proved to be in good agreement with classifying positive and negative outcomes at all possible cutoffs.

### 3.4 Proposed strategies for overcoming the impact of COVID-19 pandemic

In line with the efforts of many higher education institutions in addressing the mental and emotional health of their community, Tecnologico de Monterrey developed a number of strategies to provide support to the students at all academic levels. These strategies were focused on:

I. Providing adequate information
II. Improving communications and providing psychological assistance
III. Reducing boredom
IV. Making teaching and learning process more engaging
V. Creating an atmosphere of positivity and optimism

While these proposed strategies are not the main focus of this study, they provide insight and preventive measures for ensuring the emotional well-being of the students. A thorough explanation of our preliminary outcomes are provided in the discussion section.

## 4 Results

**Figure 1:**
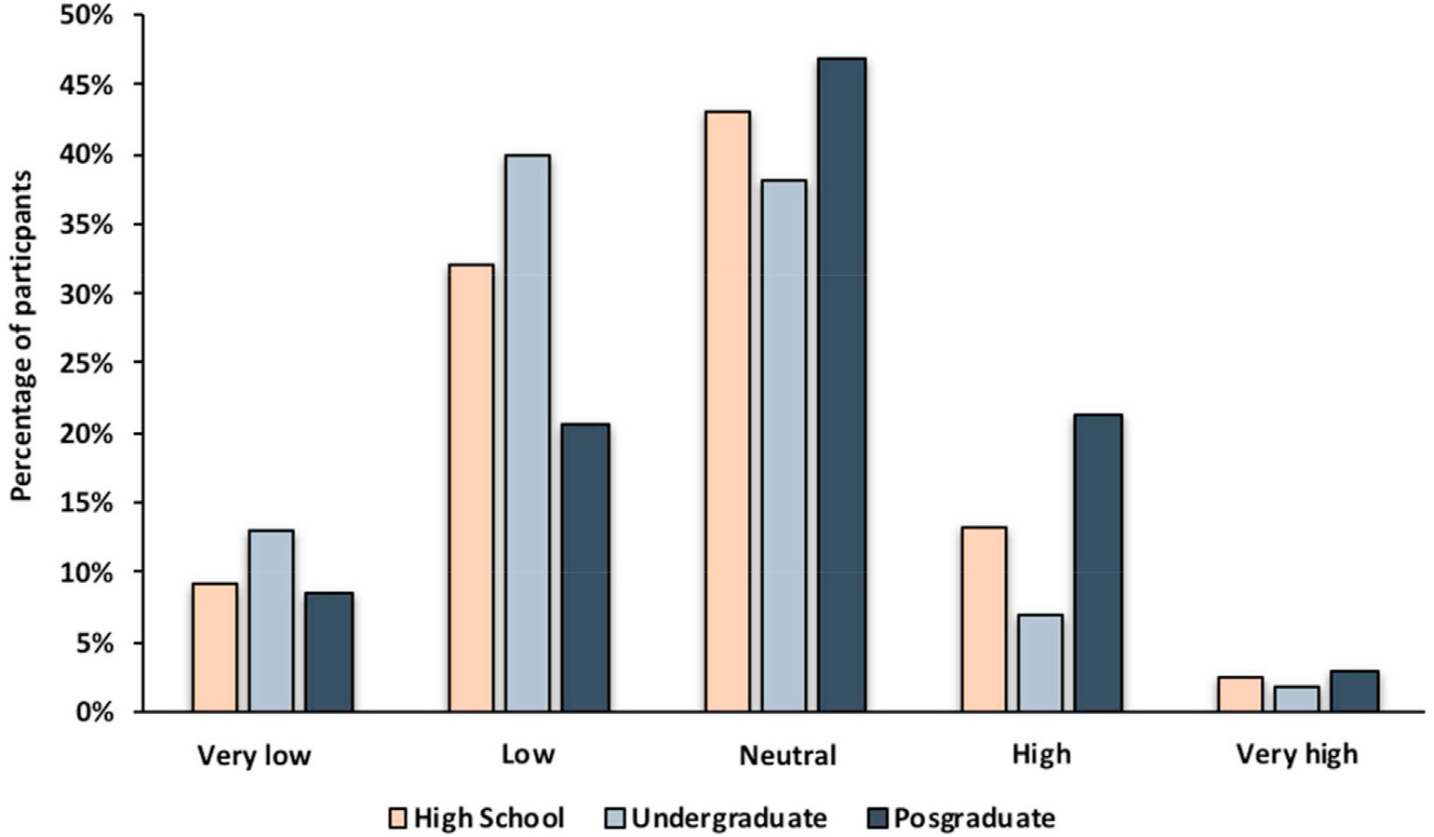
Recorded energy level of the students during W2–W8 of quarantine.

Figure 1 reports the level of energy of the students during the W2–W8 of quarantine. While majority of the HSS and the UGS seem to have felt neutral, low, or very low energy levels, the UGS, in general, have shown lower levels of energy than the HSS. Meanwhile, with an almost symmetrical distribution on both sides of ‘neutral’, the PGS have evidently felt higher levels of energy compared to the rest.

With respect to the dominant feelings of the students, Table 3 summarizes the responses of the students according to the frequency of the appearance in each academic level. Considering the emotional valence of the responses, the HSS and the UGS demonstrated mostly negative feelings with only two responses otherwise, *calm* and *thoughtful*. While for the PGS, the same two feelings were recorded (*calm* and *thoughtful*), more positive responses were recorded as well including *happy*, *thankful*, and *optimistic* (Table 3). In agreement with Figure 1, this marks another difference in the reaction of the PGS to the COVID-19 confinement compared to the HSS and the UGS. While in the first week of the remote learning most of the HSS reported to be *tired* (30.80%) and *bored* (9.89%), the UGS found themselves to be *tired* (17.88%) and *worried* (14.57%), and PGS have felt *worried* (24.73%) and *thoughtful* (19.78%).

**Table 3:** The most dominant feelings of the students throughout the first week (W1) of the quarantine and in the seven subsequent weeks (W1–W8).

High School	% W1–W8	% W1	Undergraduates	% W1–W8	% W1	Postgraduates	% W1–W8	% W1	Overall	%
Tired	15.28	30.80	Tired	13.44	17.88	Tired	11.65	13.74	Tired	13.53
*Cansado(a)*			*Cansado(a)*			*Cansado(a)*			*Cansado(a)*	
Stressed	12.63	1.90	Overwhelmed	13.20	1.10	Worried	11.33	24.73	Stressed	11.92
*Estresado(a)*			*Agobiado(a)*			*Preocupado(a)*			*Estresado(a)*	
Overwhelmed	11.07	1.14	Stressed	12.45	0.88	Thoughtful	10.70	19.78	Overwhelmed	11.20
*Agobiado(a)*			*Estresado(a)*			*Pensativo(a)*			*Agobiado(a)*	
Anxious	6.72	0.76	Anxious	9.57	1.55	Stressed	10.22	1.65	Anxious	8.40
*Ansioso(a)*			*Ansioso(a)*			*Estresado(a)*			*Ansioso(a)*	
Bored	5.97	9.89	Worried	8.06	14.57	Anxious	8.40	1.65	Worried	8.20
*Aburrido(a)*			*Preocupado(a)*			*Ansioso(a)*			*Preocupado(a)*	
Calm	5.77	0.00	Disappointed	6.84	13.25	Overwhelmed	8.00	1.65	Thoughtful	6.69
*Tranquilo(a), sereno(a)*			*Inconforme*			*Agobiado(a)*			*Pensativo(a)*	
Worried	5.70	6.46	Thoughtful	5.28	10.15	Calm	6.42	1.10	Calm	5.07
*Preocupado(a)*			*Pensativo(a)*			*Tranquil(a), sereno(a)*			*Tranquil(a), sereno(a)*	
Thoughtful	5.30	6.46	Bored	5.23	6.62	Optimistic	4.75	1.10	Disappointed	4.84
*Pensativo(a)*			*Aburrido(a)*			*Optimista*			*Inconforme*	
Depressed	4.62	6.46	Depressed	4.67	5.96	Thankful	3.80	0.00	Bored	4.61
*Deprimido(a)*			*Deprimido(a)*			*Agradecido(a)*			*Aburrido(a)*	
Disappointed	3.94	8.37	Calm	3.77	0.00	Happy	2.69	10.44	Depressed	3.97
*Inconforme*			*Tranquil(a), sereno(a)*			*Contento, Feliz*			*Deprimido(a)*	
Sad	3.87	4.94	Happy	2.64	6.18	Motivated	2.62	0.00	Optimistic	2.82
*Triste*			*Contento, Feliz*			*Motivado(a)*			*Optimista*	
Happy	3.06	6.08	Sad	2.45	5.30	Disappointed	2.54	3.30	Happy	2.78
*Contento, Feliz*			*Triste*			*Inconforme*			*Contento, Feliz*	
Optimistic	3.06	0.00	Thankful	1.84	0.00	Nervous	2.14	4.40	Sad	2.74
*Optimista*			*Agradecido(a)*			*Nervioso(a)*			*Triste*	
Nervous	2.58	3.04	Nervous	1.79	3.97	Depressed	2.06	1.65	Thankful	2.33
*Nervioso(a)*			*Nervioso(a)*			*Deprimido(a)*			*Agradecido(a)*	
Satisfied	1.90	1.14	Optimistic	1.51	0.22	Bored	1.98	1.10	Nervous	2.12
*Satisfecho(a)*			*Optimista*			*Aburrido(a)*			*Nervioso(a)*	

**Figure 2:**
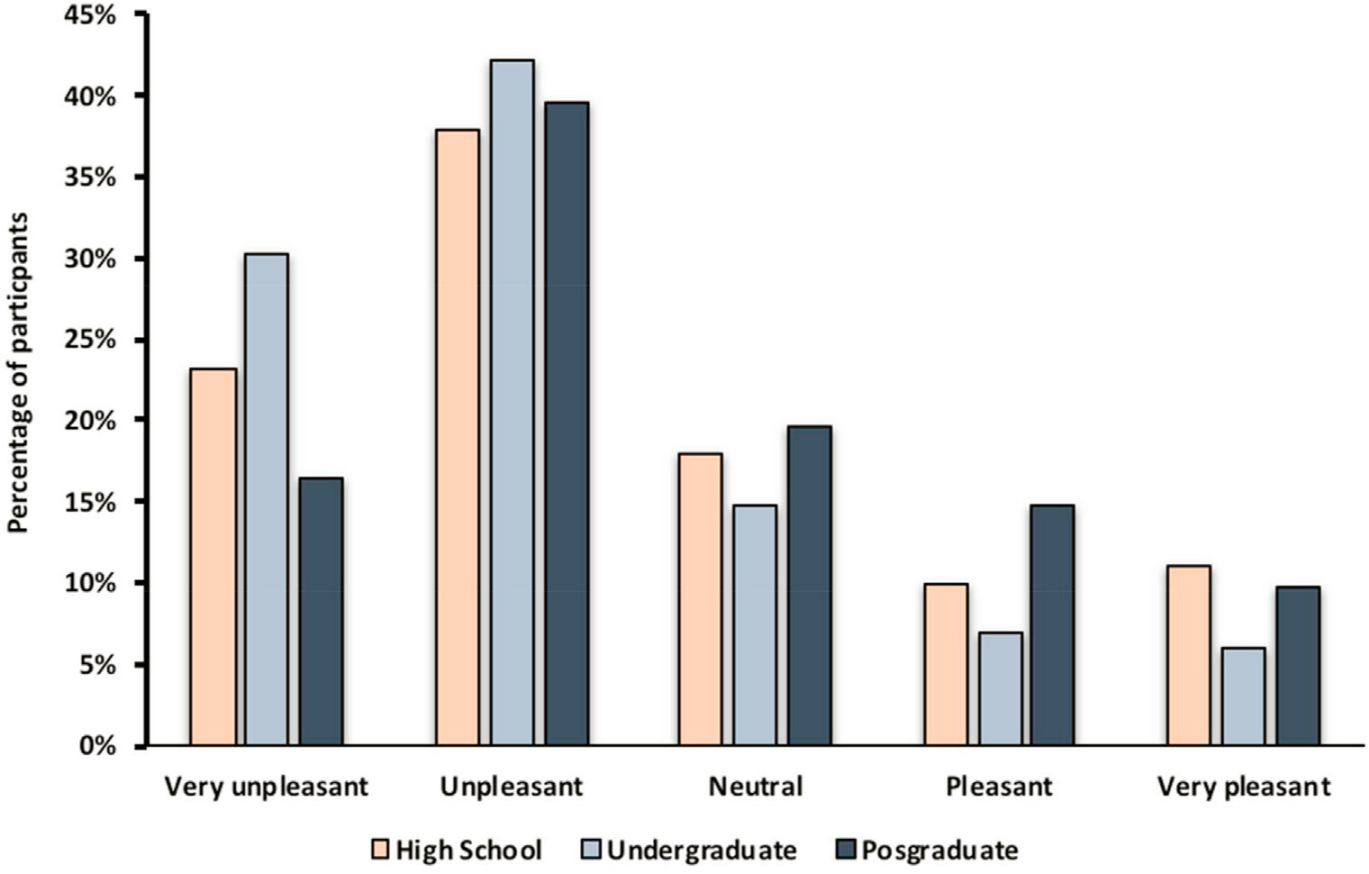
The perception of the students of their dominant feeling throughout W1–W8 of the COVID-19 pandemic.

Figure 2 represents how the students of different academic levels perceived the dominant feeling they had throughout the assessment period. The Figure shows that a vast majority of the participants perceive their dominant feelings as *unpleasant* or *very unpleasant* regardless of their educational level. However, as the graph suggests, for the HSS and the UGS, this perception seems to sharply incline towards *very unpleasant*.

**Figure 3:**
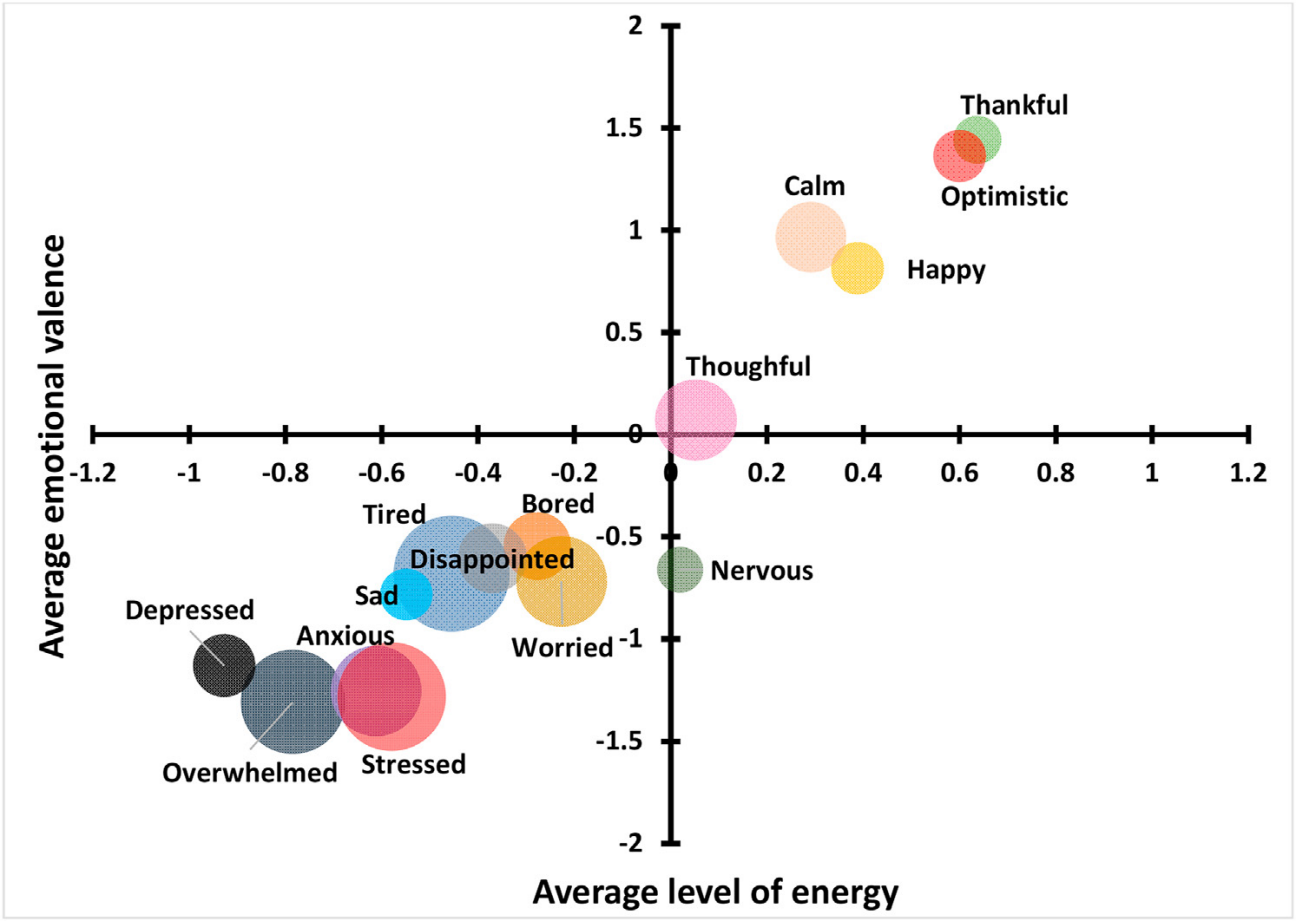
Top-fifteen feelings according to the average valence and level of energy as assessed by the participants/students. The bubbles are size-proportional to the number of counts.

The average emotional valence versus the average level of energy by main reported feelings are shown in Figure 3 with bubbles size-proportional to the number of counts. In the literature, the emotional valence is commonly classified by the researches, while in this case, it is the participants themselves who have assigned the emotional valence to their feelings by answering to Q3. Figure 3 demonstrates that for every feeling, there exists a relationship between the emotional valence and the level of energy. Negative emotional valences and low levels of energy correspond to answers including *depressed*, *overwhelmed, anxious, stressed, sad, tired, disappointed, worried, bored, and nervous*. Positive emotional valences and high levels of energy correspond to *calm, happy, optimistic, and thankful*. While *thoughtful* is seemingly in the positive emotional valence regime, it can be also considered as neutral.

**Figure 4:**
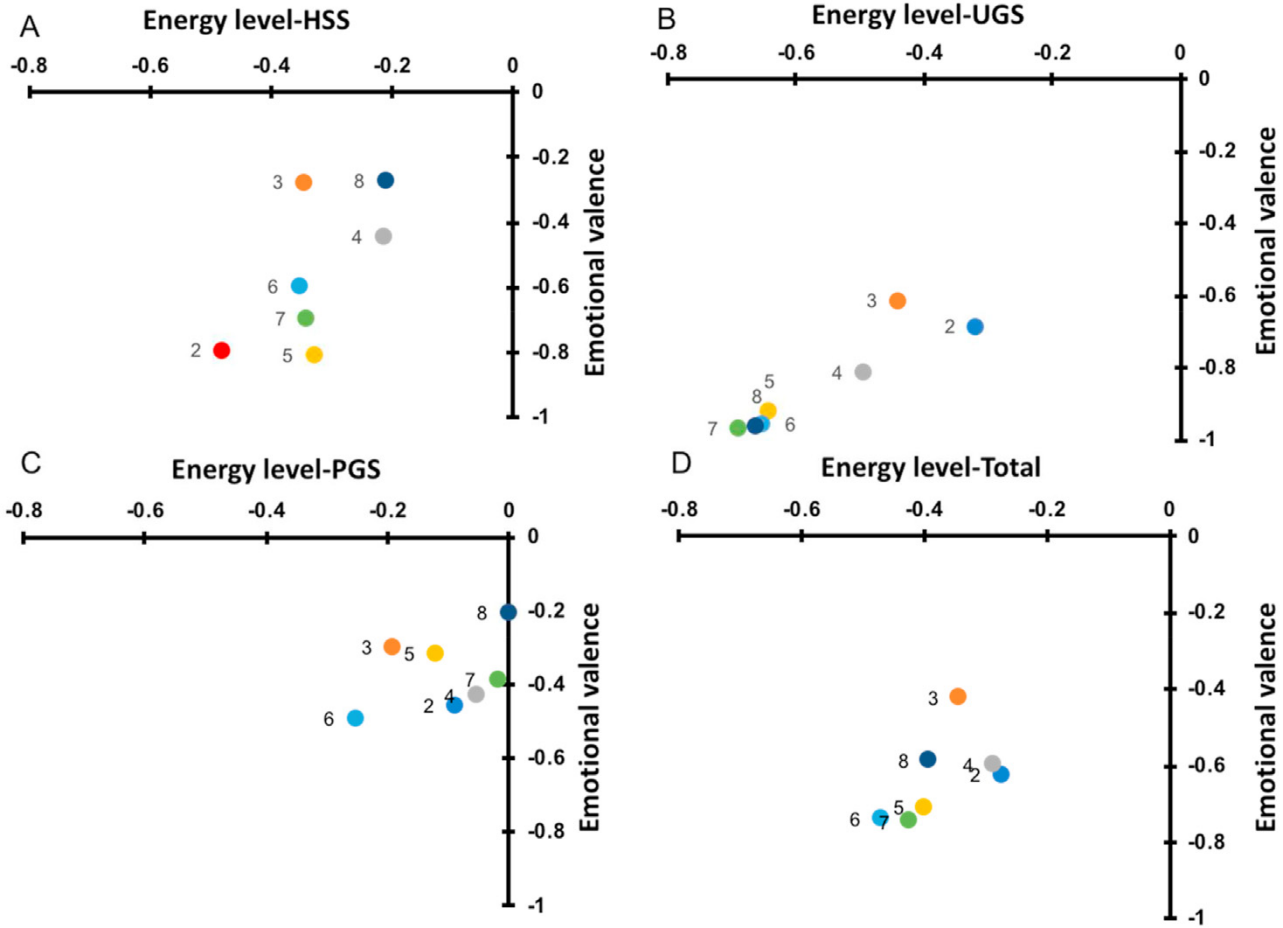
Change in the emotional valence and energy level (average values) by the weeks (represented by dots) under quarantine for A) high School, B) undergraduate, C) postgraduate, and D) total sample of students.

As the time in confinement went by, certain shifts in the students’ feelings were recorded. Figure 4 demonstrates interesting observations. The tracking of the feelings was performed in the period of W2 to W8 after the initial assessment of the feelings in W1. Figure 4A reports the feelings of the HSS with obvious emotional fluctuation as can be seen from the responses. The HSS started the quarantine at a very low emotional valence and energy level, have recuperated to occupy higher levels of both in W3 and W4, only to lose the momentum again in Weeks 5, 6, and 7. Although still negative, it is promising to record that in W8 the HSS were at a higher energy level and have recovered their emotional valence to ~ -0.3. In contrast to the fluctuating feelings of the HSS, it can be clearly seen that the UGS have systematically experienced lower energy levels and emotional valences as the time under lockdown progressed (Figure 4B). Looking into the data recorded for the PGS (Figure 4C), a general stability in both, emotional valence and energy level, can be observed which shows again that the PGS have been the least emotionally affected group of students by the pandemic circumstances. Overall, all students, regardless of their academic levels, have been feeling unpleasant and reported low energy levels (Figure 4D).

To support our findings, we also calculated the correlation coefficients for each academic level. The correlation coefficients for HSS are 0.0664 and 0.0479, for energy level and emotional valence, respectively. These numbers for PGS are 0.0241 and 0.0327, respectively. The positive values observed for both academic levels are indicative that the students acquired higher energy levels and emotional valences as the time progressed. The corresponding correlation coefficients for UGS, however, shows an opposite trend (-0.1714 for energy level and -0.1199 for emotional valence) which is in line with our previous observation (Figures 2 and 3); UGS are the only category of students who have experienced a decline in both, their energies and emotions.

**Figure 5:**
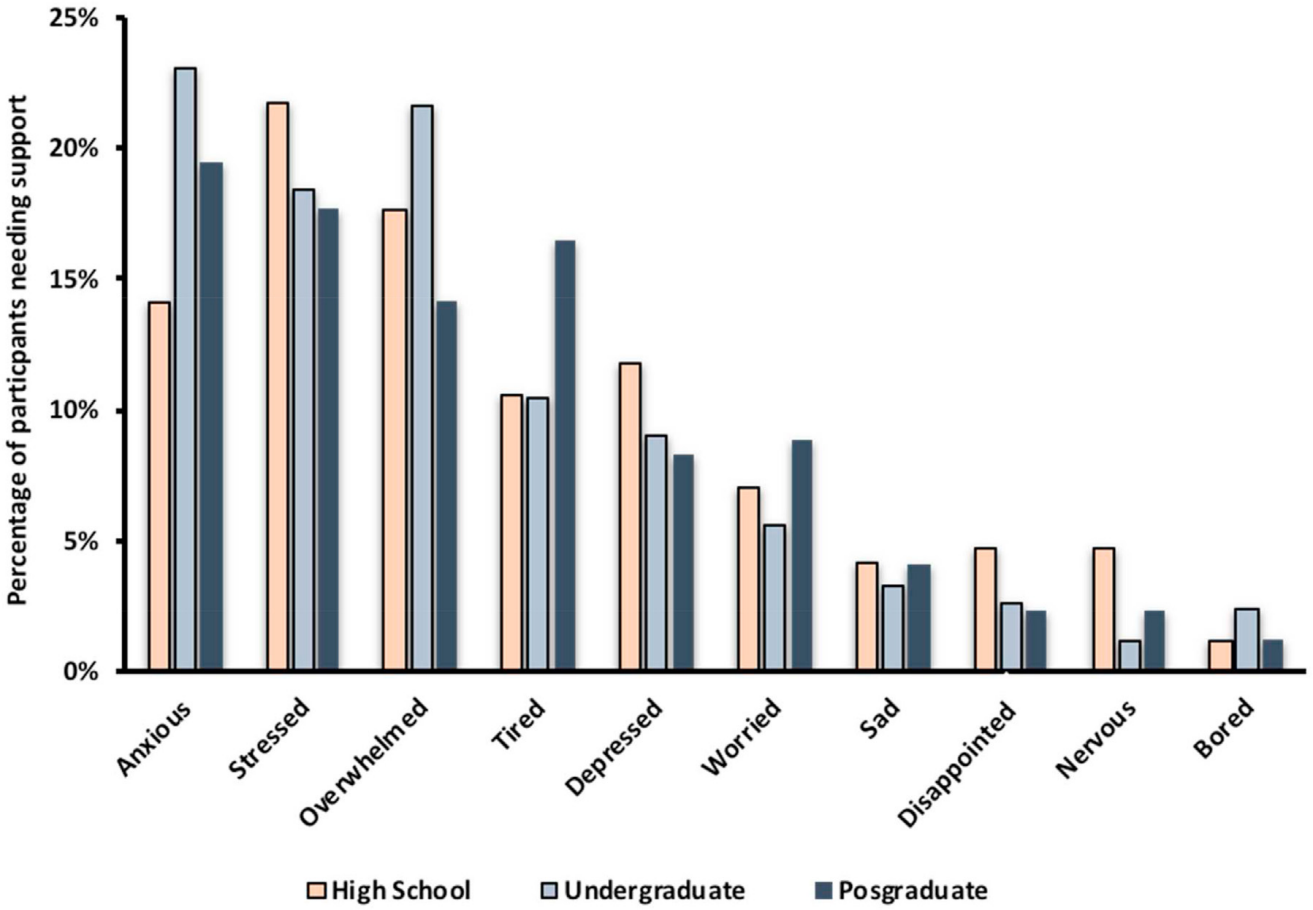
Top-ten feelings identified by the students who recognized a need for professional help to manage their feelings.

A main concern of higher education institutions during the time of pandemic is the mental health and the emotional well-being of the students. In our assessment, from the participants who answered to the survey, 11.6% of the HSS, 16.4% of the UGS, and 14.0% of the PGS identified a need for professional help to manage their feelings. The top-ten feelings reported by this specific group of students are shown in Figure 5. In an overall picture, *anxious* and *stressed* were the most frequently mentioned feelings while, as a result of the COVID-19 pandemic, the HSS have found themselves to be more *stressed* than *anxious*. Feeling *sad, disappointed, nervous*, and *bored* were also mentioned as reasons for needing professional help in less than 5% of the cases. Meanwhile, feeling *tired* was strongly indicative (16%) for PGS that they may need help. Aside from being *anxious*, feeling *overwhelmed* was the second mentioned reason why the UGS requested assistance in managing their emotions.

**Figure 6:**
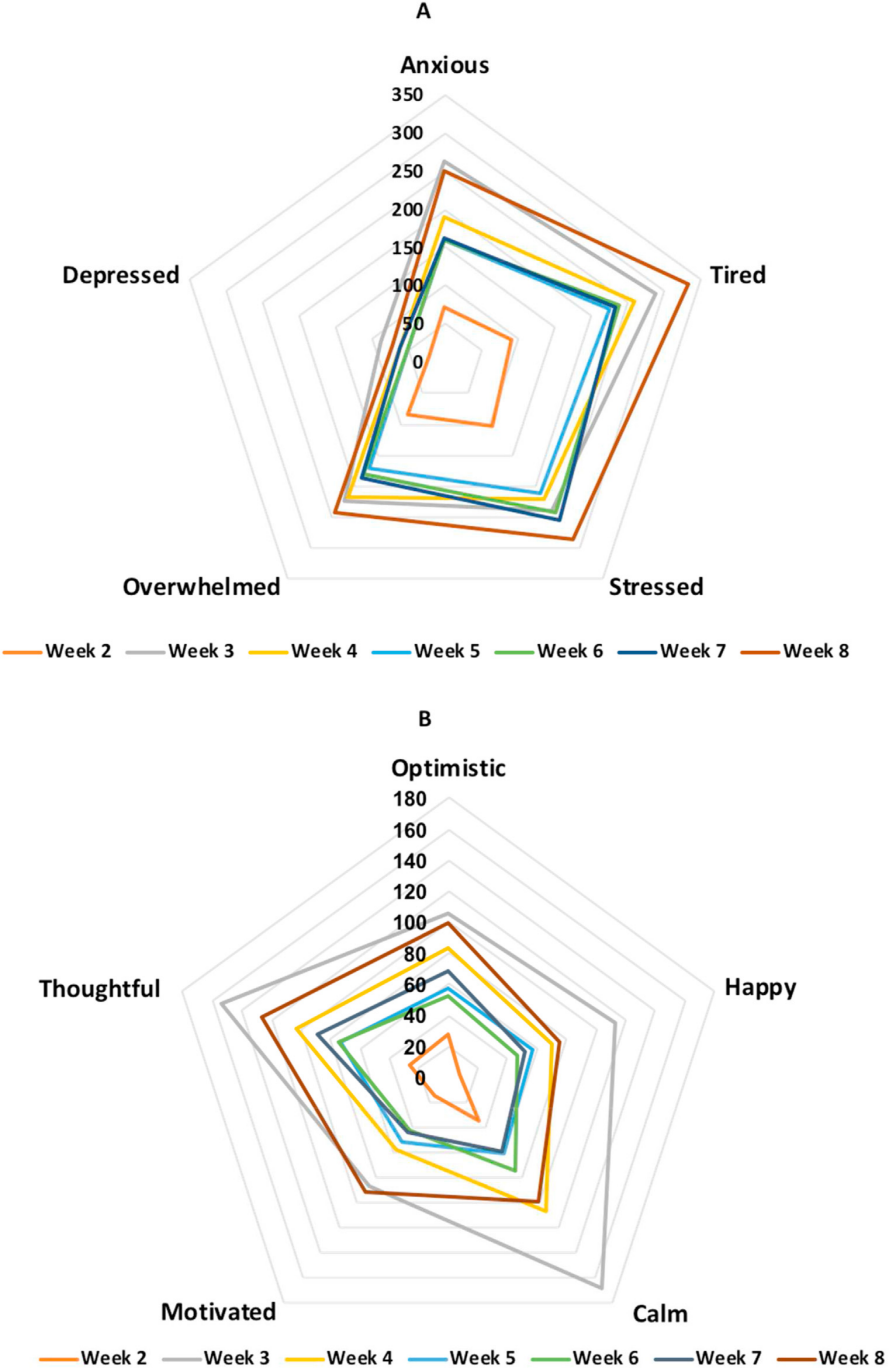
The evolution of the five most frequently reported negative (A) and positive (B) feelings of the students over W2–W8.

Figure 6 A presents the evolution of the top-five frequently mentioned negative feelings by the students who asked for professional help to manage such feelings. The top-five positive feelings and their evolution throughout the 8-week period of lockdown are also reported (Figure 6B) for comparison purposes. Except for *depressed*, which remains almost invariant as the weeks progress, the frequency of mentions in the negative feelings increases with time. Meanwhile, it is rather challenging to define a clear pattern for the positive feelings of the students over these 8 weeks (Figure 6B).

The answers to Q5 were analyzed through a multivariate logistic regression in terms of the main feelings, the level of energy, the educational level, and the number of weeks under quarantine and the results are shown in Table 4. This Table classifies the highest contributing factors to asking for professional help in two parts: the upper section represents the feelings and the lower part shows other involving parameters such as the energy and educational levels, the week of the assessment, and the intercept. The *p*-value (≤0.05) presents the relevant contributing factors to the need for professional help. Based on our analysis, while the dominance of *depressed, angry and anxious* plays the most vital role in the need for professional help in managing one's emotions, the energy level is a key playing element as well. The negative coefficient of the intercept demonstrates that a vast majority of the students (86%) were less likely to require professional help in managing their feelings.

**Table 4:** Summary of multivariate logistic regression analysis∗ for the probability of professional help requested by students of all academic levels. The upper part of the table reports the feelings and the lower part corresponds to the intercept, the level of energy, the weeks under quarantine, and the educational level (arranged based on the ascending order of the coefficient).

Variable	Scale	Coefficient	Standard Error	*p*-value
Happy	0, 1	-0.5362	0.0509	0.00642
Bored	0, 1	-0.4536	0.0479	0.00013
Calm	0, 1	-0.2883	0.0498	0.06704
Thoughtful	0, 1	-0.2850	0.0481	0.00635
Tired	0, 1	-0.2131	0.0485	0.01405
Worried	0, 1	0.1412	0.0480	0.07683
Sad	0, 1	0.1892	0.0474	0.08914
Anxious	0, 1	0.3766	0.0472	0.00005
Angry	0, 1	0.4832	0.0476	0.00612
Depressed	0, 1	0.5943	0.0487	0.00001
Intercept		-0.0704	0.0463	0.06415
Level of energy^@^	-2, -1, 0, 1, 2	-0.2253	0.0505	0.00011
Week^@@^	1, 2, 3, …, 8	0.0520	0.0504	0.01444
Educational level^@@@^	0, 1, 2	0.0849	0.0474	0.09606

@ -2, -1, 0, 1, 2 correspond to a level of energy very low, low, neutral, high, very high, respectively. @@ corresponds to the number of weeks taking online classes in quarantine. @@@ (0, 1, 2) correspond to high school, undergraduate, and postgraduate students, respectively. ∗ Regularized regression with AUC 0.702.

## 5 Discussion

This study represents one of the first efforts to investigate the feelings, the level of energy, the emotional valence, and the need for professional help in managing emotions among students at high school, undergraduate, and postgraduate levels throughout the COVID-19 pandemic. With an open-ended design of the survey, the students had the chance to report their feeling which have corresponded to 49 different items bearing mostly negative emotional valences. After having analyzed the feelings of a large number of high school students in comparison to the established feelings lists such as Positive and Negative Affect Schedule (PANAS) (Ebesutani et al., 2012), Moeller et al. (2020) highlighted the importance of having an open-ended survey where students can express their feelings in a greater detail (Moeller et al., 2020). In line with their approach, the open-ended answers to Q2, in this study, are further enriched by the use of the Spanish language and by the way the feelings relate to the perceived level of energy (Q1) and the *pleasant/unpleasant* characteristic (Q3).

Among the 15 most frequently mentioned feelings, only 1 (*calm*) had positive emotional valence in the case of the HSS and the UGS, and 4, in the case of the PGS (*calm*, *happy*, *thankful*, and *optimistic)*. Similar records of few positive feelings (5% for happiness and 4% for relief) were also collected from those quarantined due to severe acute respiratory syndrome (SARS) in 2002 (Brooks et al., 2020). We have observed that throughout W1 undergoing MFD+ and W2 under confinement, *worried* was the most frequently mentioned feeling for the PGS and the second most mentioned feeling for the UGS. This may reflect their state of awareness of the global crisis related to the pandemic and the fact that they may have a wider range of responsibilities in life (Brooks et al., 2020). Meanwhile for the HSS, the most frequently mentioned feeling was *tired* (reported by 15.28% of the participants) which agrees with a previous work reported by Moeller et al. (2020) that made the same observation for students at high school level outside the frame of a global pandemic (Moeller et al., 2020).

On the contrary to previous reports, where the emotional valence was assigned by the researchers, this work has given the freedom to the students to assign the emotional valence to their dominant feelings by selecting from *very unpleasant/unpleasant/neutral/pleasant/very pleasant* (graded as -2, -1, 0, 1, 2, respectively). We have recorded that for all participants the main reported feeling was unpleasant regardless of the academic levels they represent. While a high prevalence of negative feelings was observed for all groups of students during W1–W8, for the HSS and the UGS the emotional valence leaned towards *very unpleasant*, while the PGS identified their feeling as *unpleasant*. The responses to the level of energy and emotional valence were in good agreement; negative feelings bring low levels of energy and emotional valence, while the opposite is also true.

One purpose of this research was to address the mental health issues of the students through asking them specifically if they required professional help to manage their *unpleasant/very unpleasant* feelings. Those who have given an affirmative response to the aforementioned question were *anxious*, *stressed, overwhelmed, tired*, and *depressed*. These 5 feelings were the most frequently-mentioned in all academic levels. Among these 5 feelings, the appearance of *tired* as a reason to ask for professional help is perhaps surprising, since such feeling does not commonly appear in established scales of academic feelings reported in the literature (Ebesutani et al., 2012). Tiredness has been reported as the most frequently experienced feeling by high school students in United States as a result of sleep deprivation ("National sleep foundation-sleep in america poll-highlights and key findings," 2006). It was also identified to have detrimental impacts on students’ abilities to concentrate, learn, and to regulate their feelings (Owens and Group, 2014). We assume the reports of feeling *tired* in the time of COVID-19 pandemic also attributes to the uncertainty of the confinement and an unclear end to the current crisis. Nevertheless, feeling *anxious,* and *depressed* represents confident factors for asking for professional help (Table 4).

### 5.1 Proposed strategies to battle the undesirable consequences of COVID-19 pandemic

As the time under the quarantine restrictions progresses, it is more probable for the students to develop negative feelings (especially anxiety) which is in line with the valid concern of the educational institutions with respect to the mental well-being of their students (Baloran, 2020; van der Zanden et al., 2018). The reports of the literature suggest that having a member of family infected with COVID-19 strongly escalates the level of anxiety in students. In contrast, living with parents, having a stable family income, and living in urban areas are protective factors against anxiety (Cao et al., 2020). While our records of *anxiety* and *depression* is comparatively lower than the existing reports of the literature (Grubic et al., 2020; Torales et al., 2020; Wang et al., 2020), we have designed a comprehensive set of programs to overcome the inevitable effects of the COVID-19 pandemic and assist our students in the challenges they face. Below, we present a brief description of the outcomes of these programs while suggesting potential strategies to contribute to sustainable risk management.

VI. Providing adequate information

In the time of epidemics and global pandemics, fear of infection and infecting those around is a common concern (Rubin et al., 2016). Exposure to false information can, in turn, generate lack of clarity and further enhance the anxiety about the nature of the illness and the right preventive measures. A primary task of educational institutions, therefore, is to raise awareness and to expand the understanding of the students of the state of the SARS-CoV-2 widespread. In line with this priority, a new bilingual website was immediately established upon quarantine by Observatory of Educational Innovation within Tecnologico de Monterrey which was responsible for supplying essential information to the students and their families while rigorously filtering the data to remove the false information from the reliable and valid sources (Educativa, 2020). Since April 2020, the Spanish website has received over 225,000 views from all across LATAM with the highest number of users from Mexico, Argentina, Peru, and Colombia among others. The English website, on the other hand, distributed information among over 18,000 users from every corner of the world including India, Philippines, Bangladesh, Nigeria, and United States among others.

VII. Improving communications and providing psychological assistance

A key priority in overcoming the negative effects of the quarantine is effective communication and remaining active within one's social circles. The life in isolation and the inability to be connected to others are associated to abrupt anxiety and symptoms of long-term distress (Manuell and Cukor, 2011; G James Rubin, Brewin, Greenberg, Simpson and Wessely, 2005). Creating programs (e.g. life@home) that involve virtual socializing, and hang-out sessions are essential to keep students away from anxiety. Additionally, studies suggest that support telephone lines staffed by mental health experts can be highly effective for those in quarantine (Brooks et al., 2020; Maunder et al., 2003). “TQueremos” (We Care for You), with a 24/7 telephone line/online chat and available bilingual consultants and psychologists further assisted the students to communicate their problems, health issues, emotional challenges, personal loss, or loss of loved ones (TQueremos, 2020). Only at the high school level, the number of calls received by “TQueremos” have grown 65% since March 2020, while this number at undergraduate level reached 73%. The topics of the conversations were covering financial, legal, emotional, nutritional, and medical aspects among others. Moreover, benefiting from evidence-based practices including RULER can greatly impact the students' mental health at all academic levels through expressing and communicating their feelings (Nathanson et al., 2016). Noteworthy, Tecnologico de Monterrey has implemented the RULER strategy among high school students for the past four years. The RULER program has been an additional means for supporting students throughout the COVID-19 pandemic.

VIII. Reducing boredom

The time of lockdown and isolation generates boredom in people regardless of their age range (Brooks et al., 2020). Moeller et al. reported ~26% of the high school students to be bored. Similarly, boredom came to our attention as one of the top-15 negative feelings that the students have experienced in the time of lockdown. Another beneficial feature of life@home was over 1,200 online activities including 64 artistic, and sport online events, in addition to games that have highly engaged the students of all academic levels. Our assessment shows that 58% of the students who participated in life@home activities found them contributing and/or highly contributing to their emotional health and mental well-being. Such initiatives not only promote the spirit of togetherness and unity among students, but also make enduring the pandemic circumstances easier for them.

IX. Making teaching and learning process more engaging

Forming a collaborative network among faculty, administrators, and students and using online and information technologies facilitated a better teaching and learning experience and allowed implementing innovation in teaching. The “Budy Academico” or *Academic Buddy* program which was launched upon quarantine granted a smooth transitioning of the faculty members and students to the remote methods of teaching and learning. A great number of trained staff have relentlessly worked with over 9,000 faculty members and teachers across all high schools and university campuses throughout the first few weeks of the lockdown to insure the MFD+ was fully functional and the classes were engaging and beneficial to the students of all academic levels. As one of the core pillars of the MFD+, *Boost Your skills* program complemented the learning outcomes through platforms such as EdX and Coursera, MOOCs as well as webinars which were entirely freely available to the students during the time of the pandemic. This has generated a massive interest and a significant hike in the number of participants from January to June 2020 covering the pre-Covid-19 and period of Covid-19 outbreak. A great increase in the number of learners was observed from March to April (+41%) and May (+37%) marking ~80% of the usage across the data.

X. Creating an atmosphere of positivity and optimism

Different initiatives and activities including mindfulness and positive thinking have proven to enhance students’ resilience to stress and anxiety during challenging periods (Galante et al., 2018). Recent reports demonstrated that under lockdown, undergraduate students have decreased their physical activities and developed less healthy lifestyles (Gallè et al., 2020). In such longsome circumstances where the lack of sufficient physical activities may translate to mental and emotional exhaustion, it is important for higher education institutions to actively promote health behaviors and practices among students (Baloran, 2020). “Cuida Tu Mente” (Take Care of Your Mind) has dynamically promoted its activities including sharing positive thoughts, meditation and mindfulness, and other suitable practices throughout the lockdown period and have since become part of the daily routines of the students of all academic levels. With over 90,000 users, the program has successfully engaged participants in 114 webinars and garnered the attention of over 5,000 students to the open classes offered by the program. “Cuida Tu Mente” offers courses including *Connected Mind* (~4,000 participants), *Positive Mind* (~8,000 participants), and *Healthy Mind* (~18,000 participants) (CuidaTuMente, 2020). The results of our initial assessment showed that the students found such initiatives as strong contributing factors to their mental well-being and supportive measures to endure the life in isolation.

## 6 Conclusion

COVID-19 has taken an international toll on students' mental well-being with serious emotional consequences. These undesirable circumstances combined with health-related fears and uncertainty towards future have brought a wave of negative feelings including frustration, boredom, tiredness, anxiety, stress, depression, and anger to students of all academic levels across the world. It is, therefore, crucial for the educational institutions to remain aware of the student's mental health and to take timely actions to assist them in addressing the issues associated with COVID-19 pandemic. The current study reports a thorough analysis of the students and the spectra of their feelings at the time quarantine to assess their level of energy, their dominant feelings, and the need for professional help in managing emotions. Our findings demonstrate that students at all academic levels (high school, undergraduate, and postgraduate) demonstrated negative feelings and low energy levels as a result of the lockdown. An open-ended survey allowed us to detect a wide range of feelings experienced by the students from which 5 feelings were selected as the most notable negative feelings including anxiety, depression, tiredness, stress, and overwhelm. Overall, 14% of those who have reported these feelings and in particular found themselves to be *anxious* and/or *depressed* also recognized a need for professional help in managing their emotions. All categories of students, regardless of their academic levels, have reported low energy levels and identified the lockdown circumstances *unpleasant* or *very unpleasant*. While the students at high school level have shown fluctuating emotions over the course of 8-week analysis, the undergraduate students steadily lowered in their energy levels and the emotional valence of their feelings, while postgraduate students remained calm and composed when compared to the rest. We identified that the weeks under quarantine and the reported energy levels are key playing factors to students' mental well-being. While the results presented in this article generate valuable insights, they might not be fully representative of the students' feelings as (i) only randomly selected students from the large number of students at Tecnologico de Monterrey have answered the survey in a volunteer manner, and (ii) those who find themselves positive and with higher levels of energy would less likely to participate in a survey of such nature. The article also reports several initiatives which were designed to further assist students in overcoming the challenges of life under lockdown. In that perspective, we propose several strategies for reducing the anxiety level including (i) providing adequate information; (ii) improving communications and providing psychological assistance; (iii) reducing boredom; (iv) making teaching and learning process more engaging; and (v) creating an atmosphere of positivity and optimism.

## Declarations

### Author contribution statement

Samira Hosseini: Contributed reagents, materials, analysis tools or data; Wrote the paper.

Luis Pego: Conceived and designed the experiments.

Jose Escamilla: Contributed reagents, materials, analysis tools or data.

Claudia Camacho-Zuñiga: Analyzed and interpreted the data; Wrote the paper.

### Funding statement

This research was supported by Tecnologico de Monterrey, Mexico. The authors would like to acknowledge the financial and the technical support of Writing Lab, the Institute for the Future of Education for this production.

### Data availability statement

The data that has been used is confidential.

### Declaration of interests statement

The authors declare no conflict of interest.

### Additional information

No additional information is available for this paper.
